# Dimethyl Fumarate Alleviates Adult Neurogenesis Disruption in Hippocampus and Olfactory Bulb and Spatial Cognitive Deficits Induced by Intracerebroventricular Streptozotocin Injection in Young and Aged Rats

**DOI:** 10.3390/ijms232415449

**Published:** 2022-12-07

**Authors:** Ewelina Kurowska-Rucińska, Jan Ruciński, Dorota Myślińska, Beata Grembecka, Danuta Wrona, Irena Majkutewicz

**Affiliations:** Department of Animal and Human Physiology, Faculty of Biology, University of Gdansk, Wita Stwosza 59, 80-308 Gdansk, Poland

**Keywords:** dimethyl fumarate, sporadic Alzheimer’s disease, streptozotocin, adult neurogenesis, neuroprotection, spatial memory impairment, age, olfactory bulb, hippocampus

## Abstract

The disorder of adult neurogenesis is considered an important mechanism underlying the learning and memory impairment observed in Alzheimer’s disease (AD). The sporadic nonhereditary form of AD (sAD) affects over 95% of AD patients and is related to interactions between genetic and environmental factors. An intracerebroventricular injection of streptozotocin (STZ-ICV) is a representative and well-established method to induce sAD-like pathology. Dimethyl fumarate (DMF) has antioxidant and anti-inflammatory properties and is used for multiple sclerosis treatment. The present study determines whether a 26-day DMF therapy ameliorates the disruption of adult neurogenesis and BDNF-related neuroprotection in the hippocampus and olfactory bulb (OB) in an STZ-ICV rat model of sAD. Considering age as an important risk factor for developing AD, this study was performed using 3-month-old (the young group) and 22-month-old (the aged group) male Wistar rats. Spatial cognitive functions were evaluated with the Morris water maze task. Immunofluorescent labelling was used to assess the parameters of adult neurogenesis and BDNF-related neuroprotection in the hippocampus and OB. Our results showed that the STZ-ICV evoked spatial learning and memory impairment and disturbances in adult neurogenesis and BDNF expression in both examined brain structures. In the aged animals, the deficits were more severe. We found that the DMF treatment significantly alleviated STZ-ICV-induced behavioural and neuronal disorders in both age groups of the rats. Our findings suggest that DMF, due to its beneficial effect on the formation of new neurons and BDNF-related neuroprotection, may be considered as a promising new therapeutic agent in human sAD.

## 1. Introduction

Alzheimer’s disease (AD) is a progressive neurodegenerative disorder and the most common type of dementia (60–80% of the cases). The sporadic form of Alzheimer’s disease with late onset (sAD), where symptoms occur after the age of 65 years, affects over 95% of AD patients. sAD has a complex etiology that is still not fully understood. It is believed that sAD is not hereditary and its development is related to interactions between genetic, environmental and metabolic factors [1]. The most common diagnostic marker in AD is episodic memory impairment; however, the decline in this memory system correlates with age, and is also a symptom of other types of dementia with a different etiology (e.g., frontotemporal dementia, vascular dementia), which significantly complicates and delays diagnosis [2,3]. Recent studies suggest that the impairment of spatial memory, including spatial navigation and orientation, may be a more specific feature of AD (even in the early stages of the disease) that distinguishes AD from other types of age-related dementia and memory deficits [4,5].

Disturbances in adult neurogenesis have been proposed as an important mechanism underlying cognitive decline (including memory impairment) observed in age-related neurodegenerative disorders such as AD and, to a limited extent, in normal aging [6,7]. In the brain of adult mammals, there are only two regions where new neurons are generated. The subgranular zone (SGZ) of the dentate gyrus (DG) is the neurogenic niche for hippocampal neurogenesis, and the subventricular zone (SVZ) is a region where new neurons are formed and then migrate through rostral migratory stream to the olfactory bulb (OB). Newly generated neurons are incorporated into existing neural circuits of the hippocampus (in the DG and hippocampal subregions CA1–CA3) and play an important role in hippocampus-dependent learning and memory (e.g., spatial navigation, spatial memory and orientation). Moreover, adult hippocampal neurogenesis ensures and maintains the functional integrity of this structure, especially in aged individuals, which allows for the formation of new memories, thereby increasing the efficiency of contextual memory, and facilitates learning [6,8]. By contrast, new neurons in the OB play a critical role in perception and memory processes related to odours, which in adults may be associated with behavioural adaptive mechanisms in response to environmental changes. It has been shown that patients with mild cognitive impairment (MCI) and olfactory deficits are more likely to develop AD [9,10]. The adult neurogenesis can be regulated by many intrinsic and extrinsic factors. Growth factors and neurotrophins, such as brain-derived neurotrophic factor (BDNF), stimulate neurogenesis, increasing the proliferation, differentiation, maturation and survival of newly formed cells [11]. On the other hand, oxidative stress, neuroinflammation, insulin signalling disorders and age-related changes have been shown to inhibit neurogenesis by reducing neural stem cell proliferation and neuronal differentiation, as well as by delaying maturation and reducing the survival of new neurons [12,13,14].

Currently, there are only six drugs for the treatment of AD that have been approved by the US Food and Drug Administration (FDA) [15]. The statistics show that over 90% of the tested substances with the potential to alleviate the symptoms of AD in animal models have not completed clinical trials (or have not been approved at all). One of the reasons for such a high failure rate in clinical trials may be the selection of inappropriate animal models for preclinical studies, mainly reflecting the familial, hereditary form of Alzheimer’s disease (fAD). The animal model of an intracerebroventricular injection of streptozotocin (STZ-ICV) is one of the best characterised and clinically applicable methods to induce sAD-like pathology. Behavioural and neurochemical changes occurring in patients with sAD (e.g., cognitive impairments, insulin signalling disorders, oxidative stress, neuroinflammation, neurodegeneration, Aβ and hyperphosphorylated tau pathology) have also been found with the STZ-ICV model. Moreover, in STZ-ICV animals, the severity of cognitive and neurochemical impairments correlates with age, and such a relationship is also common in human sAD [1,16,17,18,19].

None of the pharmacological treatments available today for AD are able to slow or stop the neurochemical changes leading to neurodegeneration that causes AD symptoms and makes the disease fatal. Moreover, they are often associated with decreasing efficacy over time and undesirable side effects. Unfortunately, the discovery of new drugs (design, in vitro studies, animal model testing, clinical trials) requires an average of 15 years and huge financial resources before a therapeutic agent is available to patients. Furthermore, many of the new drugs do not even reach the market, despite all the time and cost invested in research and development. Therefore, the application of established drug compounds to new therapeutic interventions (drug repurposing) may be a solution to this problem.

Dimethyl fumarate (DMF) has antioxidant, anti-inflammatory and neuroprotective properties. In 2013, an oral drug Tecfidera, in which DMF is an active compound, was registered as the primary therapy for patients with relapsing–remitting multiple sclerosis (RRMS) due to its high neuroprotective and immunomodulatory efficacy, a satisfactory level of safety in prolonged treatment and a positive balance of possible benefits and side effects [20,21]. One of the main mechanisms responsible for the antioxidant properties of DMF is its influence on the nuclear factor erythroid 2-related factor 2 (Nrf2) signalling pathway. The Nrf-2 target genes control the cell’s redox homeostasis, energy metabolism, DNA repair processes, cell survival and proliferation. The anti-inflammatory and immunosuppressive actions of DMF occur mainly through the inhibition of the nuclear factor kappa-light-chain-enhancer of the activated B cells (NF-κB) pathway [22]. Recently, the ability of DMF to increase BDNF production has also been confirmed in several studies: after spinal cord injury [23], in a hypothyroidism rat model [24] and in a D-galactose/ovariectomy-induced rat model of AD [25].

In our previous studies, we found that DMF therapy prevented the STZ-ICV-induced disruption of spatial memory, loss of Ch1 cholinergic cells, hippocampal interleukins 6 and 10 induction and neurodegeneration in 4-month-old rats [17]. We also showed that the STZ-ICV acts age dependently, as the aged animals (22-month-old rats) had greater spatial memory impairments, neurodegeneration, microglia activation and oxidative stress in the hippocampus. We confirmed that DMF therapy reduces age-dependent microglia activation and consequently limits oxidative stress and neurodegeneration in STZ-ICV rats [18].

The present study aims to determine whether DMF therapy attenuates the disruption of adult neurogenesis in two neurogenic regions—the DG and OB in the STZ-ICV rat model of sAD. The influence of DMF on adult neurogenesis has not been studied so far using an in vivo model of sAD. Furthermore, the present study examines the level of neuroprotection associated with BDNF, which is known to promote neurogenesis and the survival of neurons. Moreover, the present study aims to determine whether the effect of both the STZ-ICV and DMF treatment on the assessed parameters is age dependent, considering the fact that age is one of the most important risk factors for developing AD. For this purpose, cognitive processes, adult neurogenesis and BDNF-related neuroprotection were assessed in two age groups of rats: young (3-month-old) and aged (22-month-old) animals.

## 2. Results

### 2.1. DMF-Containing Chow or Standard Chow Intake, Daily DMF Dose and Body Weight

The results of the average DMF-containing chow intake and average body weight of the rats at the beginning of and on the last day of the experiment are shown in Table 1.

To determine whether the chow intake corresponds to the DMF dose that was effective in other studies, we measured the daily chow intake, calculated daily DMF intake (we used chow containing 0.4% DMF by weight) and next calculated the dose for each rat subjected to the DMF treatment based on its final body weight (Table 1). The result showed that the average daily DMF dose was 44 mg/kg for all the animals receiving chow with DMF. Body weight reduction during the experiment reached an average level of 5% in the young STZ+DMF rats, less than 1% in the young Sham+DMF rats (in this group, the increasing body weight was found in four animals) and 8% in both (STZ+DMF and Sham+DMF) aged rats (Table 1).

### 2.2. Spatial Learning and Memory—MWM Test

The screening trial revealed that all the rats were able to find and climb the visible platform within 60 s, so the behavioural analyses were performed on all the rats.

#### 2.2.1. Spatial Learning—Acquisition of Long-Term Memory

**Figure 1:**
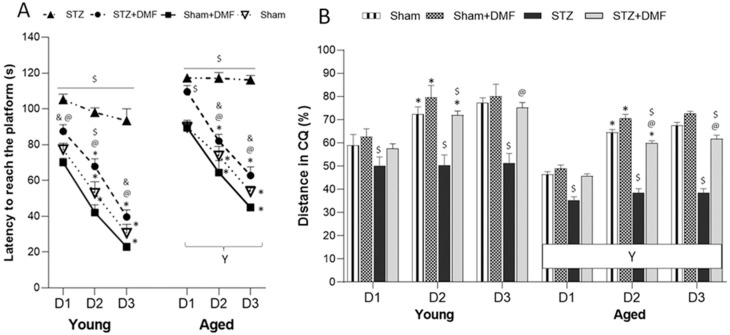
Effects of STZ-ICV and DMF treatment on spatial learning (3 days of sessions, 4 trials per session, platform position fixed) evaluated by Morris water maze in young (3-month-old) and aged (22-month-old) rats. (**A**) Latency to reach the platform in 1–3 sessions (D1, D2, D3) in the MWM. (**B**) Percentage of total distance travelled in the critical quadrant (CQ) in 1–3 sessions in the MWM. Data are expressed as mean ± SEM of trials; *n* = 10 animals/group. Explanations: $: *p* < 0.05 vs. both Sham groups within the age group; &: *p* < 0.05 vs. only Sham+DMF within the age group; @: *p* < 0.05 vs. STZ within the age group; *: *p* < 0.05 vs. previous day within the experimental group; Y: *p* < 0.05 young vs. aged rats within the experimental group by three-way ANOVA followed by Tukey’s post-hoc test.

The results of the spatial learning (acquisition of long-term memory) expressed as latency to reach the platform and the percentage of the total distance travelled in the critical quadrant (quadrant of the maze with the platform, CQ), which were evaluated in the sessions 1–3 of the MWM test, are shown in Table 2 and Figure 1. A four-way ANOVA with repeated measures revealed that all the tested factors, sAD-like model, DMF therapy, age and day of the test, significantly influenced both measured parameters. There were also significant interactions between those factors (Table 2).

Tukey’s post-hoc test showed that the animals in the STZ group presented spatial learning deficits when compared to the Sham groups (Sham and Sham+DMF receiving an ICV of the citrate buffer) in both age groups. Moreover, the aged STZ rats took the longest time to complete the task (latency to reach the platform) (Figure 1A) and had the lowest percentage of total distance travelled in the CQ (Figure 1B). Importantly, both the young and aged STZ rats were unable to improve their spatial learning skills from day to day—Tukey’s post-hoc test showed no significant reduction in latency to reach the platform and no significant increase in the percentage of the distance travelled in the CQ in day 2 and day 3 when compared to the previous day (Figure 1A,B).

Spatial learning deficits were alleviated in the STZ rats with DMF therapy. The young STZ animals treated with chow containing DMF needed significantly less time to reach the platform each day of the spatial learning task in the MWM as compared to the young STZ rats receiving standard chow (Figure 1A). In the aged STZ+DMF rats, this pattern was found on day 2 and 3 of the MWM (on day 1, the STZ+DMF rats also showed shorter latency as compared to the STZ group, but this difference was statistically insignificant) (Figure 1A). Each day of the spatial learning task, both the young and aged STZ+DMF rats travelled a significantly higher % of the total distance in the CQ as compared to the STZ animals of the same age (Figure 1B). Importantly, the DMF therapy also improved the dynamic of the spatial learning as both age groups of the STZ+DMF animals showed a significant reduction in latency to reach the platform from day to day (Figure 1A). A statistically significant increase in the percentage of the distance travelled in the CQ from day to day was found in both the young and aged STZ+DMF rats between day 2 and day 1 of the test (Figure 1B). This pattern was similar to that of the control rats (Sham and Sham+DMF). In addition, Tukey’s post-hoc test revealed only a few significant differences between the animals in the STZ+DMF and Sham groups in both age groups, which may indicate that the DMF treatment led to an almost complete normalisation of STZ-ICV-induced spatial learning deficits (Figure 1A,B).

Moreover, both the young and aged Sham+DMF rats had a better spatial learning performance in latency when compared to the STZ+DMF rats of the same age on each day of the test. Differences between the STZ+DMF and Sham+DMF rats in the % of the total distance travelled in the CQ were statistically significant on day 2 in the young and on day 2 and 3 in the aged animals (Figure 1A,B).

The aged rats showed a significantly worse spatial learning performance in both measured parameters compared to the young animals each day in the MWM test in all experimental groups (Figure 1A,B).

#### 2.2.2. Reference Memory Performance

**Figure 2:**
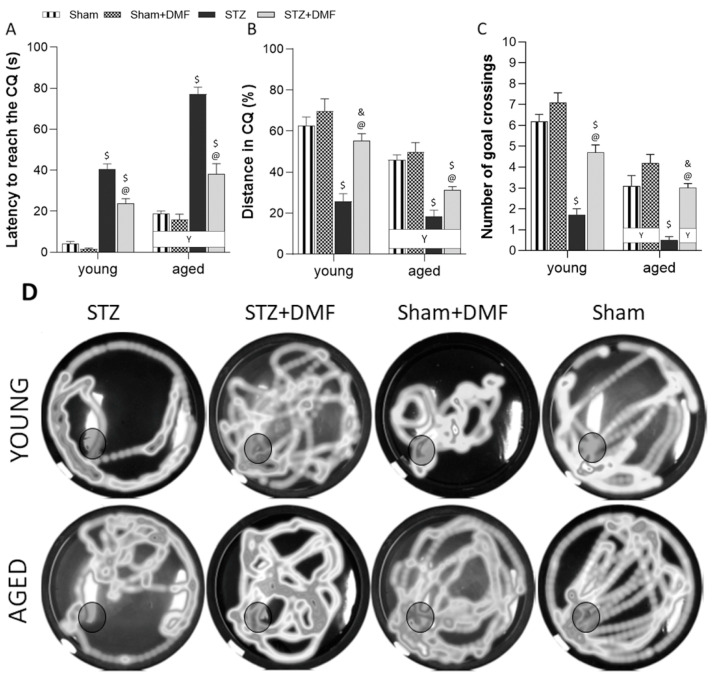
Effects of STZ-ICV and DMF treatment on reference memory performance in probe test evaluated by Morris water maze in young (3-month-old) and aged (22-month-old) rats. (**A**) Latency to reach the critical quadrant (CQ) in which the platform was located in the previous sessions of spatial learning in the MWM. (**B**) Percentage of the total distance travelled in the CQ. (**C**) Number of goal crossings indicates how often an animal crossed the former platform position. (**D**) Visualisation of the path shape and the number of goal (black circle) crossings of representative rat of each group. Data (**A**–**C**) are expressed as mean ± SEM; *n* = 10 animals/group. Explanations: $: *p* < 0.05 vs. both Sham groups within the age group; &: *p* < 0.05 vs. only Sham+DMF within the age group; @: *p* < 0.05 vs. STZ within the age group; Y: *p* < 0.05 young vs. aged rats within the experimental group by three-way ANOVA followed by Tukey’s post-hoc test.

The results of the reference memory performance in the probe test in the MWM, assessed as latency to reach the CQ (in which the platform was in the previous sessions of spatial learning), and the % of the total distance travelled in the CQ and the number of goal crossings (which indicates how often an animal crossed the former platform position) are shown in Table 3 and Figure 2. A three-way ANOVA revealed that all the tested factors, i.e., the sAD-like model, DMF therapy and age significantly influenced all the measured parameters. There were also significant interactions between these factors (Table 3).

Tukey’s post-hoc test showed that the STZ-ICV caused a severe disruption in recalling spatial information stored in the reference memory as both the young and aged STZ rats needed significantly more time to reach the CQ (Figure 2A), travelled significantly less distance in the CQ (Figure 2B) and very rarely or not at all crossed the goal (place in the maze where the platform was located in the previous three days of the MWM test; Figure 2C,D) during the probe test compared to the control rats (Sham and Sham+DMF) of the same age.

The DMF therapy attenuated the reference memory disorders induced by the STZ-ICV in both age groups of animals. The STZ+DMF rats reached the CQ significantly faster (Figure 2A), crossed the goal more often (Figure 2C,D) and travelled a longer distance in the CQ (Figure 2B) when compared to the STZ rats. Tukey’s post-hoc test revealed significant differences between the STZ+DMF rats and both control groups (Sham and Sham+DMF) in almost all the measured parameters; therefore, it can be assumed that the reference memory was not fully restored by the DMF therapy in both the young and aged rats (Figure 2).

Significant age-related differences in all the experimental groups were found only in latency to reach the CQ—the aged rats needed significantly more time to reach the CQ compared to the young animals (Figure 2A). For the remaining parameters, such a relationship was observed in the STZ+DMF animals and control groups (Sham and Sham+DMF) (Figure 2B–D).

#### 2.2.3. Working Memory Performance

**Figure 3:**
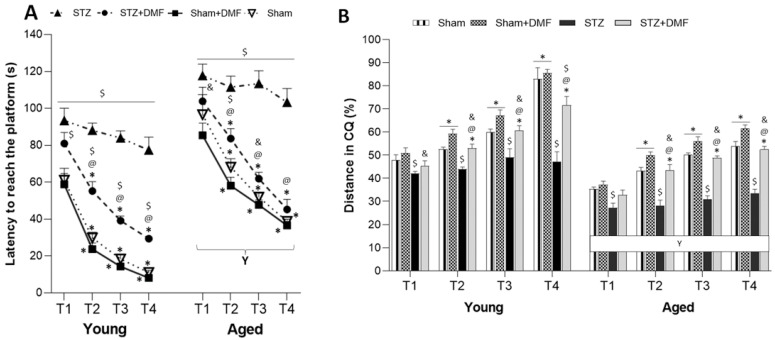
Effects of STZ-ICV and DMF treatment on working memory (3 days of sessions, 4 trials per session, platform position changed daily) evaluated by Morris water maze in young (3-month-old) and aged (22-month-old) rats. (**A**) Latency to reach the platform in 4 trials (T1–T4) during the sessions 5 7 in the MWM. (**B**) Percentage of the total distance travelled in the CQ in 4 trials (T1–T4) during the sessions 5–7 in the MWM. Data are expressed as mean ± SEM of days; *n* = 10 animals/group. Explanations: $: *p* < 0.05 vs. both Sham groups within the age group; &: *p* < 0.05 vs. only Sham+DMF within the age group; @: *p* < 0.05 vs. STZ within the age group; *: *p* < 0.05 vs. previous trial within the experimental group; Y: *p* < 0.05 young vs. aged rats within the experimental group by three-way ANOVA followed by Tukey’s post-hoc test.

The results of the working memory performance expressed as latency to reach the platform and the percentage of the total distance travelled in the CQ, which were evaluated in the sessions 5–7 of the MWM test, are shown in Table 4 and Figure 3. A four-way ANOVA with repeated measures revealed that all the tested factors, i.e., the sAD-like model, DMF therapy, age and trial of the test significantly influenced both measured parameters. There were also significant interactions between these factors (Table 4).

In the working memory task, which lasted three days with the platform’s position changing daily, the animals had to scan the maze to find the platform in the first trial, while in subsequent trials (2–4), the rats used the acquired spatial information about the platform’s location stored in short-term memory. Tukey’s post-hoc test revealed that the STZ-ICV led to working memory disorders. The young and aged animals in the STZ group took a significantly longer time to reach the platform (Figure 3A) and travelled less distance in the CQ (Figure 3B) each trial in the MWM compared to the control rats (Sham and Sham+DMF) of the same age. The dynamics of working memory functioning was also disrupted by the STZ-ICV, as the STZ animals showed no significant reduction in latency and no significant increase in the % of distance in the CQ from trial to trial (Figure 3A,B).

The DMF therapy improved the working memory performance in both age groups of animals. The young and aged STZ+DMF rats needed significantly less time to reach the platform (Figure 3A) and travelled a significantly longer distance in the CQ (Figure 3B) as compared to the STZ rats of the same age in trials 2–4. In the first trial, these differences were not statistically significant. Importantly, due to only one significant difference between the aged STZ+DMF and aged Sham rats (latency, trial 2), it can be assumed that DMF therapy in the aged rodents with the STZ-ICV led to the complete normalisation of working memory in both measured parameters. In the young rats, such a relationship (lack of significant differences between STZ+DMF and Sham, except trial 4) was observed only in the distance travelled in the CQ (Figure 3B), while differences in the latency between these groups were statistically significant in each trial. However, both the young and aged STZ+DMF rats presented a similar dynamic of working memory functioning to that found in the control animals (Sham and Sham+DMF) (Figure 3A,B).

Moreover, there were significant differences between the STZ+DMF and Sham+DMF groups in the latency (each trial for the young animals and 1–3 trials for the aged rats) and % of the total distance travelled in the CQ (except trial 1 for the aged animals): the young and aged Sham+DMF rats presented a better working memory performance compared to the STZ+DMF rats of the same age (Figure 3A,B).

The aged rats in all the experimental groups showed significantly worse working memory performance in both measured parameters compared to the young animals during each trial in the MWM test (Figure 3A,B).

### 2.3. Adult Neurogenesis

**Figure 4:**
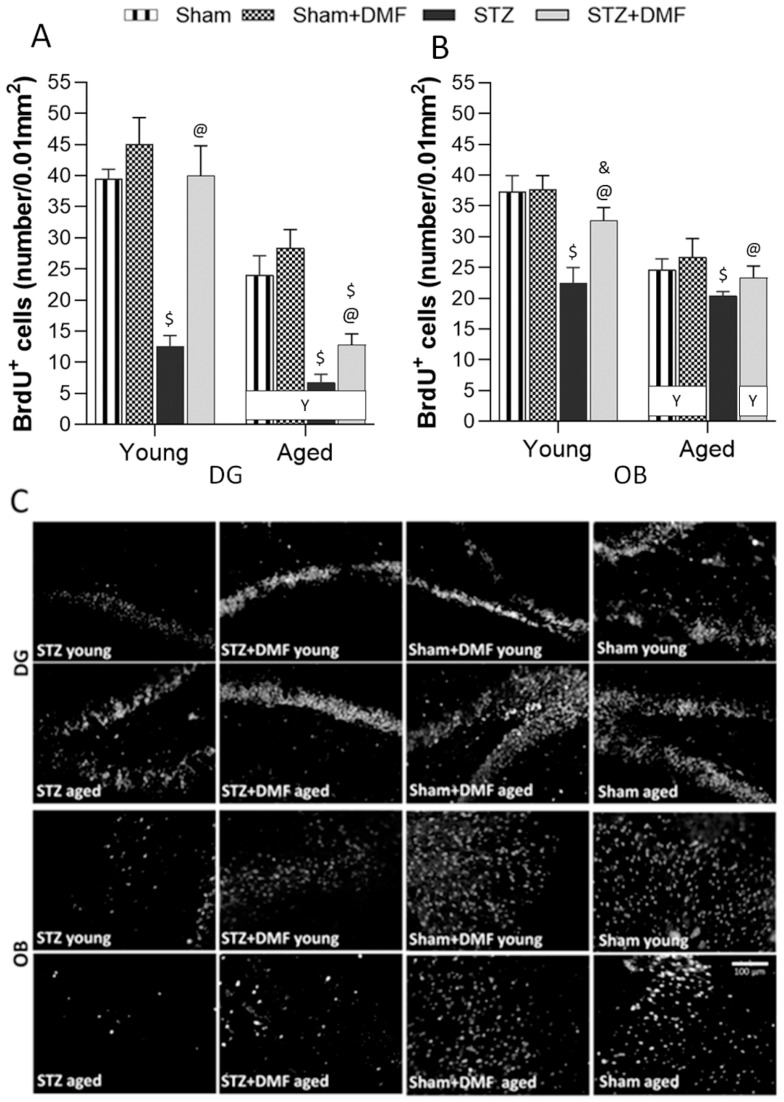
Effects of STZ-ICV and DMF treatment on neural proliferation in young (3-month-old) and aged (22-month-old) rats. Quantification of BrdU-containing cells in the dentate gyrus of the hippocampus (**A**) and in the olfactory bulb (**B**). (**C**) Representative photomicrographs of BrdU-labelled cells in the DG and OB of young and aged animals of all experimental groups. Scale bar = 100 μm. Data (**A**,**B**) are expressed as mean ± SEM; *n* = 10 animals/group. Explanations: $: *p* < 0.05 vs. both Sham groups within the age group; &: *p* < 0.05 vs. only Sham+DMF within the age group; @: *p* < 0.05 vs. STZ within the age group; Y: *p* < 0.05 young vs. aged rats within the experimental group by three-way ANOVA followed by Tukey’s post-hoc test.

**Figure 5:**
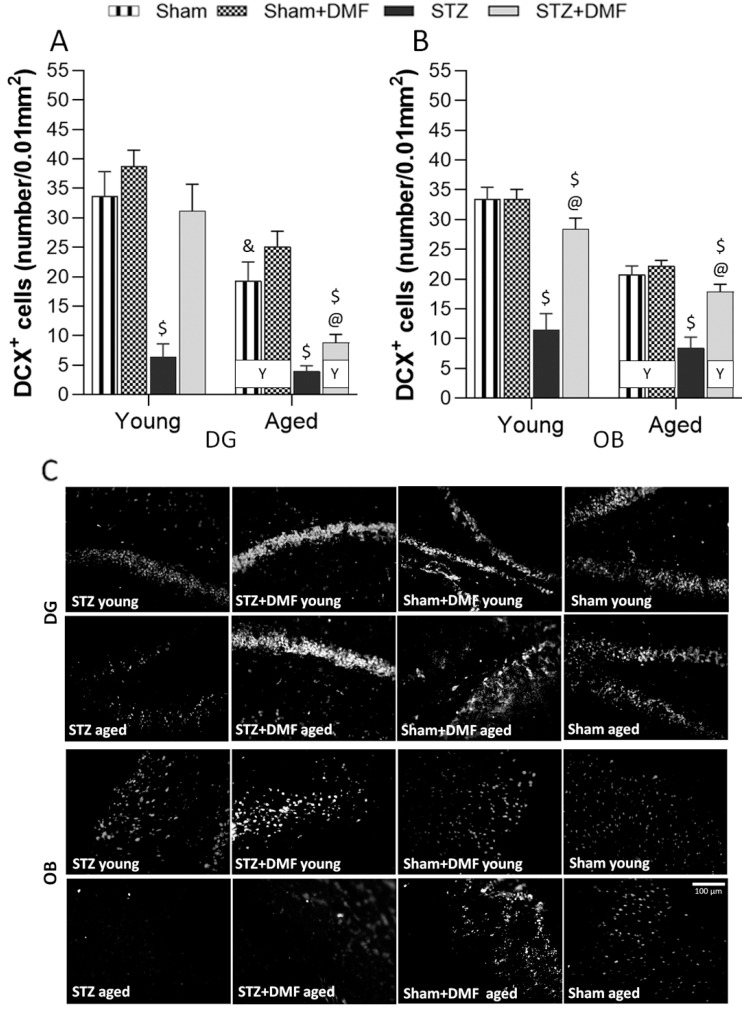
Effects of STZ-ICV and DMF treatment on neuronal differentiation in young (3-month-old) and aged (22-month-old) rats. Quantification of DCX-containing cells in the dentate gyrus of the hippocampus (**A**) and in the olfactory bulb (**B**). (**C**) Representative photomicrographs of DCX-labelled cells in the DG and OB of young and aged animals of all experimental groups. Scale bar = 100 μm. Data (**A**,**B**) are expressed as mean ± SEM; *n* = 10 animals/group. Explanations: $: *p* < 0.05 vs. both Sham groups within the age group; &: *p* < 0.05 vs. only Sham+DMF within the age group; @: *p* < 0.05 vs. STZ within the age group; Y: *p* < 0.05 young vs. aged rats within the experimental group by three-way ANOVA followed by Tukey’s post-hoc test.

**Figure 6:**
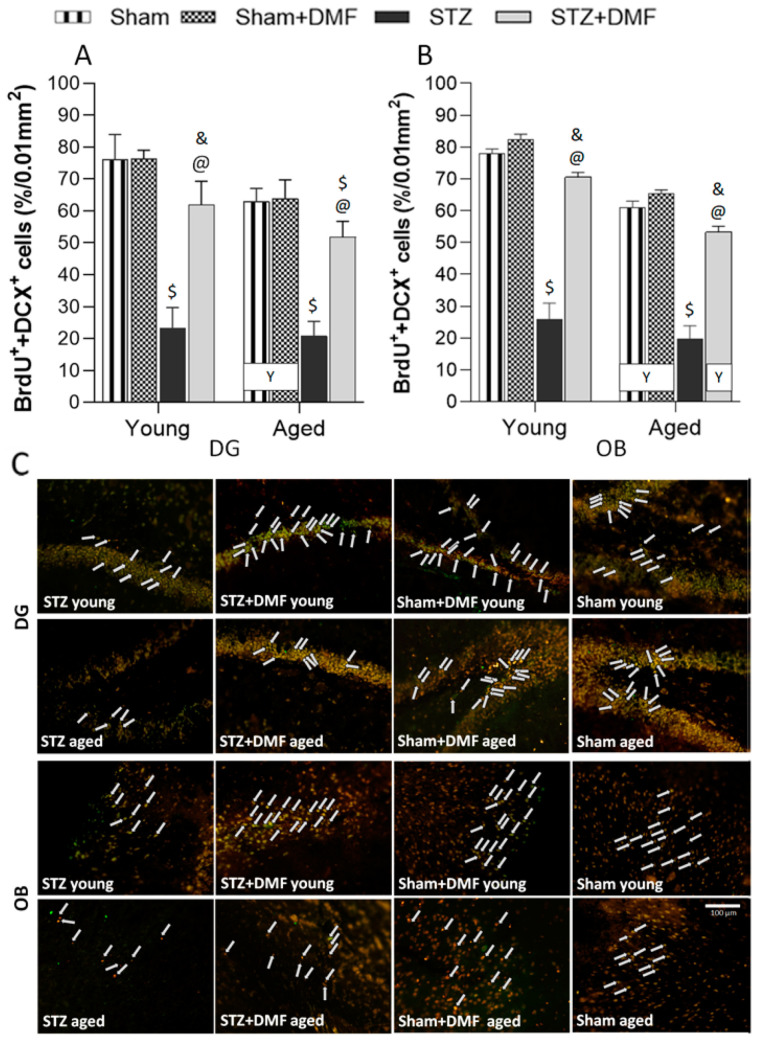
Effects of STZ-ICV and DMF treatment on adult neurogenesis level in young (3-month-old) and aged (22-month-old) rats. Percentage of double-labelled cells (BrdU+DCX-containing cells—newly formed immature neurons) of the sum of all fluorescent cells in the dentate gyrus of the hippocampus (**A**) and in the olfactory bulb (**B**). (**C**) Representative photomicrographs of BrdU+DCX-labelled cells in DG and OB of young and aged animals of all experimental groups. Double labelled (BrdU+DCX) cells are indicated by white arrows. Scale bar = 100 μm. Data (**A**,**B**) are expressed as mean ± SEM; n = 10 animals/group. Explanations: $: *p* < 0.05 vs. both Sham groups within the age group; &: *p* < 0.05 vs. only Sham+DMF within the age group; @: *p* < 0.05 vs. STZ within the age group; Y: *p* < 0.05 young vs. aged rats within the experimental group by three-way ANOVA followed by Tukey’s post-hoc test.

The results of the neural cells proliferation (quantification of BrdU-containing cells), differentiation (quantification of DCX-containing cells) and the level of the newly formed immature neurons (percentage of BrdU+DCX-containing cells of the sum of all fluorescent cells) in the DG of the hippocampus and OB are shown in Table 5 and Figure 4, Figure 5 and Figure 6. A three-way ANOVA revealed that all the tested factors, i.e., the sAD-like model, DMF therapy and age significantly influenced all the measured parameters of adult neurogenesis. There were also significant interactions between these factors (Table 5).

#### 2.3.1. Neural Cells Proliferation and Neuronal Differentiation

Tukey’s post-hoc test showed that the STZ-ICV impaired proliferation (Figure 4) and differentiation (Figure 5) in the DG and OB in the young and aged STZ animals compared to the Sham groups (Sham and Sham+DMF) of the same age. The aged STZ rats presented the lowest number of new cells (BrdU-containing cells) and developing immature neurons (DCX-containing cells). In both age groups of the STZ animals, the number of cells containing BrdU (Figure 4) as well as DCX (Figure 5) was higher in the OB when compared to the DG.

The DMF therapy significantly attenuated the effect of the STZ-ICV on neural proliferation and neuronal differentiation, as the DG and OB of the young and aged STZ+DMF animals were characterised by significantly more new cells (Figure 4) and immature neurons that survived (Figure 5) compared to the STZ rats of the same age.

Moreover, the number of BrdU-positive cells and DCX-positive cells in the DG of the young STZ+DMF animals was not significantly different as compared to the young Sham rats (Sham+DMF and Sham groups). In the DG of the aged STZ+DMF rats, the number of new cells (Figure 4A,C) and immature neurons (Figure 5A,C) was significantly lower compared to both Sham groups of the same age. The OB of both age groups of the STZ+DMF animals presented lower number of BrdU-containing cells as compared to the Sham rats of the same age, but only the difference between the young STZ+DMF animals and young Sham+DMF rats was statistically significant (Figure 4B,C). The number of DCX-containing cells in the OB was significantly lower in both the young and aged STZ+DMF animals compared to the Sham+DMF and Sham group rats of the same age (Figure 5B,C).

Age-related differences were found in both neurogenic regions of the STZ+DMF rats and control rats (Sham+DMF and Sham) as the young animals presented a higher number of new cells (Figure 4) and neuronal developing cells (Figure 5) compared to the aged animals, and only in the DG of STZ rats in the number of BrdU-containing cells.

#### 2.3.2. Level of the Newly Formed Immature Neurons

Tukey’s post-hoc test showed that the STZ-ICV reduced the formation of new immature neurons in both neurogenic regions (the DG and OB) in the young and aged STZ animals compared to the Sham groups (Sham and Sham+DMF) of the same age (Figure 6).

The DMF therapy also significantly improved the level of newly formed immature neurons (BdrU+DCX-containing cells) in the DG (Figure 6A,C) as well as in the OB (Figure 6B,C) of the young and aged STZ+DMF rats as compared to the STZ rats. Furthermore, it can be assumed that the DMF therapy led to the normalisation of the double labelled cells level (BrdU+DCX) in the young and aged STZ+DMF animals in the OB (Figure 6B,C) and in the young STZ+DMF rats in the DG (STZ+DMF vs. Sham differences were not statistically significant) (Figure 6A,C). The Sham rats receiving chow with DMF in both age groups had the highest level of newly formed immature neurons in the DG and OB, which was also significantly higher than that in the STZ+DMF rats (Figure 6).

The OB of the young rats in STZ+DMF and both control groups (Sham+DMF and Sham) were characterised by a higher level of the newly formed immature neurons compared to the aged animals (Figure 6B,C). In the DG, such a relationship was observed only in the control animals (Sham+DMF and Sham) (Figure 6A,C). Age-related differences in the level of cells containing both markers in the DG and OB of STZ rats were not statistically significant (Figure 6).

### 2.4. Neuroprotection—BDNF-Containing Cells

**Figure 7:**
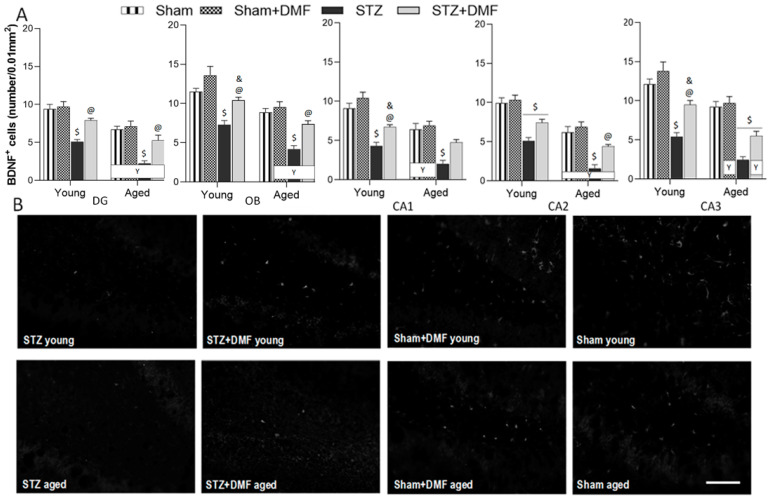
Effects of STZ-ICV and DMF treatment on neuroprotection in young (3-month-old) and aged (22-month-old) rats. (**A**) Quantification of BDNF-containing cells in both neurogenic regions: the dentate gyrus of the hippocampus and olfactory bulb, and in CA1–CA3 areas of the hippocampus. Data are expressed as mean ± SEM; young *n* = 10 animals/group, aged *n* = 8–10 animals/group. (**B**) Representative photomicrographs of BDNF-labelled cells in the DG of young animals in all experimental groups and in aged STZ and STZ+DMF rats. Scale bar = 100 μm. Data (**A**) are expressed as mean ± SEM; *n* = 10 animals/group. Explanations: $: *p* < 0.05 vs. both Sham groups within the age group; &: *p* < 0.05 vs. only Sham+DMF within the age group; @: *p* < 0.05 vs. STZ within the age group; Y: *p* < 0.05 young vs. aged rats within the experimental group by three-way ANOVA followed by Tukey’s post-hoc test.

The results of the BDNF-related neuroprotection evaluated by the quantification of BDNF-containing cells are shown in Table 6 and Figure 7. A three-way ANOVA revealed that all the tested factors, i.e., the sAD-like model, DMF therapy and age significantly influenced the number of BDNF-containing cells in all the analysed regions in the hippocampus (CA1–CA3 areas and DG) and OB. There were also significant interactions between these factors (Table 5).

Tukey’s post-hoc test showed that the STZ-ICV evoked an impairment of neuroprotective activity by reducing the number of BDNF-containing cells in both neurogenic regions as well as in CA1–CA3 areas of the hippocampus in the young and aged STZ rats compared to the control animals (Sham+DMF and Sham) of the same age (Figure 7A,B). The disruption of neuroprotection associated with BDNF was more severe in the aged STZ animals as they had the lowest number of BDNF-containing cells in all analysed hippocampal regions and in the OB (Figure 7A).

The DMF therapy significantly alleviated the BDNF-related neuroprotection impairment in the young and aged STZ+DMF rats in both neurogenic regions (DG and OB) as compared to the STZ animals of the same age (Figure 7A,B). In the CA1 and CA3 areas of the hippocampus, only the young STZ+DMF rats had a significantly higher number of BDNF-positive cells compared to the young STZ animals, and in the CA2 area, such a relationship was found only in the aged STZ+DMF rats compared to the aged STZ animals (Figure 7A). The level of neuroprotection associated with BDNF in the STZ+DMF animals was similar to that observed in the Sham animals receiving standard chow in most cases: in animals of both age groups in the CA1, DG and OB, only in the aged rats in CA2, and only in the young rats in CA3 (Figure 7A,B).

Moreover, in all the analysed regions, the Sham+DMF rats presented the highest number of BDNF-containing cells in the young as well as in the aged animals. In the OB of the young Sham+DMF rats, the level of BDNF-related neuroprotection was significantly higher compared to the young STZ+DMF rats. The same relationship was found in the CA1–CA3 areas of the hippocampus of the young animals, and in the hippocampal area CA3 in the aged rats (Figure 7A).

The age factor analysis revealed that the DG, CA2 and OB of the young STZ rats were characterised by significantly more BDNF-containing cells as compared to the aged animals. In the STZ+DMF group, this pattern was found in almost all the analysed regions, except the CA1. Age-related differences in the Sham rats were statistically significant in the DG, CA1 and CA2, whereas in the Sham+DMF group, in all the analysed brain regions, the young animals had a significantly higher number of BDNF-containing cells compared to the aged rats (Figure 7A,B).

## 3. Discussion

In this study, for the first time, the effect of DMF therapy on neural proliferation, neuronal differentiation and the number of newly formed immature neurons in the DG and OB was tested in the STZ-ICV-induced rat model of sAD. The obtained results suggest that this compound has the ability to reduce disturbances in adult neurogenesis evoked by the STZ-ICV in both examined neurogenic regions of young and aged rats. In studies with animal models of neurodegenerative diseases, the dose of DMF considered effective is in a range of 30 mg/kg to 100 mg/kg, as reviewed by Majkutewicz et al. [22]. We demonstrated that the average daily DMF dose in our study was 44 mg/kg for all the animals receiving chow with DMF (Table 1); thus, it was in line with the data reported in the literature.

Most studies involving STZ-ICV-induced animal models of sAD have reported a disruption of adult hippocampal neurogenesis, although there are few studies that have shown no such effect. Guo et al. [26] did not observe a significant effect of the STZ-ICV on the immature neurons (DCX-positive cells) in the hippocampus of young rats 2 months after the STZ-ICV. Sun et al. [27] compared the effects of the STZ-ICV on the adult hippocampal neurogenesis of young rats in short-term (1 month after the STZ-ICV) and long-term (3 month after the STZ-ICV) paradigms. No significant influence of the STZ-ICV on adult neurogenesis was found in the short-term paradigm, but in the long-term paradigm, the STZ-ICV reduced the number of mature (NeuN^+^) and immature neurons, including mitotic active neuronal cells (NeuroD^+^) and postmitotic neuronal cells (DCX^+^).

The results obtained by our team suggest that the STZ-ICV led to adult hippocampal abnormalities in the young and aged rats, which occurred 22 days after the last STZ-ICV injection, in contrast to the studies cited above. One explanation for these differences may be a specific time pattern in the severity of the STZ-ICV-induced deficits. It has been shown that dysfunctions evoked by the STZ-ICV are associated with time after the treatment, which includes three phases: (1) acute—developing within one month after the STZ-ICV; (2) a temporary improvement (complete or partial normalisation to the pre-STZ-ICV level) between 1 and 3 months after the treatment; and (3) decompensation with slow and chronic progression in the severity of the disruptions from 6 months after the STZ-ICV. This pattern of the development of cognitive and neurochemical impairment is very similar to the human AD pathology and has also not been found in transgenic animal AD models [19,28,29]. In studies that confirmed a significant impact of the STZ-ICV on adult hippocampal neurogenesis, the analyses were usually performed earlier than 1 month after the STZ-ICV or at least 3 months after the treatment. The authors demonstrated that the STZ-ICV induced a reduction in the proliferation (Ki-67^+^ or BrdU+nestin^+^), differentiation (DCX^+^), maturation (NeuN^+^) and survival (BrdU+NeuN^+^) of the neurons in the DG of rodents. These changes were accompanied by increased levels of neuroinflammatory markers and oxidative stress [13,30,31,32,33]. In the present study, we also found a lower number of BrdU-containing cells and DCX-containing cells in the DG of young and aged STZ rats, which indicates disturbances in the proliferation and differentiation of newly formed cells into a neuronal phenotype. Moreover, in our previous study, we showed that young and aged STZ animals presented a higher number of activated microglia (CD68^+^), increased level of oxidative/nitrative stress and neurodegeneration in the hippocampus compared to control rats [18].

It is considered that gliosis, a noncancerous proliferation of glial cells, may indicate the intensification of neuroinflammation, which is one of the most important pathological mechanisms in neurodegenerative diseases, including AD [1,34,35]. Another factor strongly involved in the pathogenesis of sAD is oxidative stress, which has been shown to be one of the crucial mechanisms leading to the STZ-ICV-induced disturbances [1,19]. The reactive oxygen species (ROS) are accumulated during adult neurogenesis as a physiological mechanism. However, the accumulation of high levels of ROS, usually due to overproduction or inadequate clearance, disrupts adult neurogenesis. ROS overload has been shown in neuroinflammation, neurodegeneration and ageing [14]. It is worth emphasizing that brain tissue contains high levels of lipids and has relatively low activity of enzymatic and nonenzymatic antioxidant protection; thus, it is susceptible to ROS damage, whose accumulation over time may contribute to AD pathogenesis [36].

Despite demonstrating a strong correlation of olfactory deficits and AD development [10,37], little is still known about the mechanisms underlying these disorders. In adult humans, the number of newly formed neurons in the OB is significantly lower compared to rodents. However, there is some evidence indicating that new neurons are continuously incorporated in the human OB during adulthood [38,39]. Therefore, the dysfunction of adult neurogenesis in the OB may contributes to the olfactory symptoms occurring in AD. To our knowledge, only one study evaluated the influence of the STZ-ICV on adult neurogenesis in the OB. Mishra et al. [40] showed that an STZ-ICV reduced the number of newly formed cells (BrdU^+^) in the SVZ as well as the number of immature neurons (DCX^+^) in the SVZ and OB of young rats 20 days after the STZ-ICV. Our results are in accordance with cited findings, as we also found that the STZ-ICV evoked severe disturbances in the proliferation, neuronal differentiation and survival of newly formed immature neurons in the OB of young and aged rats.

In this study, we confirmed that DMF therapy alleviates STZ-ICV-induced disturbances in adult neurogenesis in both neurogenic regions—in the DG and OB. Moreover, the DMF treatment led to complete normalisation in all the analysed parameters of neurogenesis in the DG, but only in the young animals. In the OB, complete normalisation in the proliferation (BrdU^+^) and survival of new immature neurons (BrdU+DCX^+^) was found in young and aged rats, whereas the DMF therapy did not restore the neuronal differentiation level in both age groups of animals. There is no study evaluating DMF’s influence on adult neurogenesis in vivo; however, its efficacy has been confirmed in vitro in the stimulation of the differentiation of oligodendrocyte progenitor cells [41]. Considering the fact that AD is a multiple and complex disease which results from many pathological processes that have a significant role in neuroinflammation and oxidative stress, it can be assumed that an effective therapeutic agent should exhibit several equally strong mechanisms of action. The antioxidant and anti-inflammatory properties of DMF have been well established, and approved drugs in which DMF is a primary therapeutic compound are currently used in the treatment of psoriasis and RRMS. The therapeutic potential of DMF has also been reported in cellular (in vitro) and rodent models of several neurodegenerative diseases [22,42]. In our previous studies, we showed that DMF therapy improved a broad spectrum of disturbances evoked by an STZ-ICV in a rat model of sAD. The DMF treatment ameliorated the loss of cholinergic cells in the medial septum projecting to the hippocampus, level of the neuroinflammatory markers, oxidative/nitrative stress and neurodegeneration in the hippocampus of rats subjected to the STZ-ICV [17,18]. Several mechanisms of the DMF action (or its metabolite—monomethyl fumarate) have been described, and they may play an important role in the attenuation of least some of the pathological conditions occurring in AD. The beneficial effect of the DMF treatment on adult neurogenesis confirmed in this study may be associated with a significant impact of this compound on Nrf2-dependent and NF-κB-dependent signalling pathways. DMF, by activating Nrf2, stimulates antioxidant enzyme production in many cells, including neurons [43], as well as the expression of the genes involved in cell survival and proliferation [20]. It has also been shown that DMF stimulates signal transduction via the PI3K/Akt pathway, which leads to phosphorylation, and thus the inhibition of GSK-3β kinase. The increased activity of GSK-3β is associated with Aβ plaques formation, tau hyperphosphorylation, the promotion of the inflammatory response and enhanced apoptosis of newly formed mature neurons [44,45].

BDNF is one of the most potent factors stimulating neurogenesis [46]; thus, another aim of our study was to determine whether the DMF treatment improved the dysfunction of BDNF-related neuroprotection. We found that the STZ-ICV led to a reduction in BDNF-containing cells in the hippocampus (DG and CA1–CA3 areas) as well as in the OB of rats in both age groups. The young and aged animals receiving the STZ-ICV and fed with DMF-containing chow had a significantly better level of neuroprotection associated with BDNF, which was similar to the control rats (Sham group) of the same age in almost all examined brain structures. A similar effect was found by Luo et al. [47], who found a decrease in the BDNF level in the hippocampus 4 weeks after the second injection of STZ (the procedure of the administration of STZ and the dose was identical to that of the present study). Oral therapy with DMF alleviated the STZ-induced deficit of BDNF, as our STZ+DMF rats showed more cells expressing BDNF than STZ rats in the hippocampus and olfactory bulb in both age groups. These results are in accordance with the study of Abd El-Fatah et al. [25], where the beneficial effect of DMF on the BDNF level in the hippocampus was found in another model of AD, resembling postmenopausal dementia. Female 18-month-old ovariectomised rats injected with D-galactose (ip) were used. Two-month oral therapy with DMF normalised the BDNF level in the hippocampus. Taking into account the above data, we expected an alleviation of the decline in BDNF expression due to DMF therapy in the AD model used. Our results on the number of BDNF-containing cells and neurogenesis are in mutual accordance.

In our study, the STZ-ICV-induced disruption of adult neurogenesis and loss in BDNF-related neuroprotection was accompanied by spatial learning and memory impairment assessed in the MWM test. We showed that the STZ rats presented the most severe cognitive deficits, including the dynamic of spatial learning and working memory performance, and recalling spatial information stored in long-term reference memory. The obtained results indicated that the DMF therapy alleviated these disturbances. Moreover, the DMF treatment led to the normalisation of spatial learning and working memory, as there were only a few significant differences in the measured parameters between the STZ+DMF and control animals (Sham group) in both age groups. The DMF therapy did not fully restore the functionality of long-term reference memory, as the young and aged STZ+DMF rats presented significantly lower levels of the measured parameters compared to the control animals (Sham group) of the same age in the probe test in the MWM.

Our results are in agreement with other studies investigating effect of the STZ-ICV on spatial cognitive processes (e.g., [33,48,49,50,51]). The cited data and results obtained in this study may have a great importance in the context of spatial cognitive deficits considered as an early behavioural marker of AD in humans. Spatial navigation is a complex cognitive function that is essential for the independence, quality of life and safety of older adults [2], and is less influenced by important confounding variables such as educational level, verbal abilities and cultural bias, unlike episodic memory [52]. The spatial disorientation in AD patients may be related to a decreased density of hippocampal neurons, especially in the CA1 and CA3 areas [53,54]. Allison et al. [55] confirmed that the wayfinding task involving spatial cognitive skills can be used to differentiate healthy older adults from patients with AD. Importantly, spatial cognitive deficits were also found in MCI patients, who are more likely to develop AD [56]. Our results together with the provided data suggest that spatial cognitive deficits may be a valuable diagnostic tool in the early stage of AD or may be used to predict AD development. The tests assessing spatial memory in humans, e.g., an analogue of the MWM [57], may enhance the translational value of animal-based research focused on developing new therapeutic strategies.

The results obtained in this study showed that the DMF treatment effectively improved spatial learning and memory deficits in rats subjected to an STZ-ICV. Similar findings were reported by Abd El-Fatah et al. [25], who found that DMF attenuated memory deficits, hippocampal neurodegeneration and astrocyte activation in ovariectomised D-galactose-injected aged female rats. In another study by Rojo et al. [58], DMF had a beneficial effect on spatial cognitive disruption in transgenic mouse models of AD with amyloidopathy and tauopathy. These mice exhibited motor deficits and a terminal spinal deformity and died prematurely, at around 14 months of age. Six weeks of therapy with DMF reduced glial and inflammatory markers and improved cognition and motor dysfunctions by stimulating NRF2 activity. Recently, Sun et al. [59] found that DMF reduced memory impairment and hippocampal atrophy induced by Aβ and ibotenate acid injections into the mice hippocampus, and that it also delayed the progression of AD by an Nrf2-dependent mechanism. So far, the results from only one study are in contrast with the results obtained by our team and the findings cited above. Möhle et al. [60] demonstrated that the DMF treatment did not ameliorate spatial memory deficits assessed in the MWM and neuroinflammatory processes in the APP/PS1 transgenic mouse model of AD. The authors justified the discrepancies of the results with previous studies by highlighting differences in the models’ characteristics, i.e., blood-brain barrier (BBB) integrity or its disruption, which can facilitate drug penetration into the brain. The STZ-ICV was shown to damage the BBB [61], whereas, in the model of AD that was similar to that used by Möhle et al. [60], there was no disruption of the BBB in 6-month-old mice [62]. However, older APP/PS1 mice (12-month-old) showed an impairment in the BBB [63]. Therefore, the age of the animals and the age of the onset of symptoms in AD can be the important factor influencing the results of DMF preclinical efficacy in various models of AD. APP/PS1 mice show an early onset of cerebral amyloidosis (between 6–8 weeks of age) [64], which does not reflect the development of sAD in humans [19,29]. The pathogenic role of the Aβ in AD is still controversial, and many researchers believe that the overload of Aβ and plaques formation may represent late events rather than a cause (as reviewed by Alves et al. [1]). Moreover, it has already been demonstrated that Aβ plaques can be present in older people without cognitive impairment, and the cognitive decline observed in patients with AD or MCI is only weakly related to the change in Aβ deposition [65,66]. These findings suggest that animal models of AD focused on Aβ as a main cause of symptom progression may be inadequate. However, further studies with other transgenic and nontransgenic models of AD evaluating DMF’s influence on spatial cognitive deficits and amyloidopathy is undoubtedly required.

Our results demonstrated that the age of the animals was an important factor modifying the effect of the STZ-ICV and DMF therapy on behavioural and neuronal changes. The aspects of age-related differences in the therapeutical effectiveness of DMF in animal models of AD is still poorly known, as most studies use animals of one age.

We confirmed that the aged animals presented worse spatial learning skills, working memory performance and recalling information stored in long-term reference memory compared to the young animals in all experimental groups. The STZ-ICV led to a more severe disruption in spatial cognitive performance, as the aged STZ rats were characterised by the worst scores in every spatial task in the MWM. The DMF treatment restored learning and memory function in both age groups of animals; however, the aged rats coped with the spatial tasks significantly worse compared to the young rats in the STZ+DMF group. Our findings are consistent with the literature data from studies on rodents and humans reviewed by Lester et al. [67] and Plácido et al. [68].

The influence of age on adult neurogenesis was also observed in this study. We found that only proliferation (BrdU^+^) was more severely disrupted by the STZ-ICV in the aged rats compared to the young STZ animals in the DG, but not in the OB. Age-related differences in neuronal differentiation and the number of newly formed immature neurons in both examined neurogenic regions in the STZ group were not statistically significant. The DMF therapy seemed to be age dependent in both neurogenic regions for all the measured parameters, as the young STZ+DMF rats presented a higher number of labelled cells compared to the aged animals, except for the number of newly formed immature neurons in the DG. This age-dependent pattern was similar to what was found in both control groups (Sham+DMF and Sham). The DMF therapy in the young and aged rats was equally effective at ameliorating adult neurogenesis disturbances evoked by the STZ-ICV in both neurogenic regions. Many studies have confirmed that adult neurogenesis in aged rodents is approximately 3–9 times lower compared to juveniles. The age-related deterioration of adult neurogenesis includes every stage of this process: the proliferation, differentiation, maturation and survival of newly formed neurons [8,69,70,71]. Age-related decline in adult human neurogenesis remains controversial [72], although Moreno-Jiménez et al. [12] showed a negative correlation between the number of immature neurons and age in healthy people. It was found that the number of neural stem cells and neural progenitors, as well as the proportion of astrocytes to neurons in the hippocampus of young and aged rats, remained the same, but significantly fewer cells were actively undergoing mitosis in aged animals. This effect may be caused by a decreased level of proteins stimulating neurogenesis, especially BDNF [73]. Our results are in agreement with these findings. We found that aged rats showed a lower number of BDNF containing cells in both neurogenic regions. A deficit of BDNF is considered one of most potent factors involved in aging processes in the hippocampus, including the disruption of neurogenesis, changes in hippocampal cytoarchitecture and expression of neurotransmitters [74,75]. Interestingly, age-related changes in microglial cells appear much faster in neurogenic zones compared to other regions of the brain, reducing the survival of newly formed neurons [76]. In our previous study, we found that microglia activation, oxidative/nitrative stress and neurodegeneration in the hippocampus were more severe in the aged rats compared to the young animals, and the DMF treatment was more effective in aged rats [18]. Considering the fact that age is one of the most important risk factors for developing AD, the results obtained in this study and our previous findings may contribute to a better understanding the role of age-related changes in the STZ-ICV model of sAD, and in the effectiveness of the DMF treatment.

## 4. Materials and Methods

### 4.1. Animals and Experimental Approach

All animal procedures were carried out in accordance with the European Communities Council Directive of 24 November 1986 (86/609/EEC) and under the authority of the Local Ethical Committee for the Care and Use of Laboratory Animals at the University of Technology in Bydgoszcz, Poland.

Male Wistar rats (*n* = 80) were used in our study. The animals were divided into two age groups: 3-month-old rats (the young group, *n* = 40) weighing 300 ± 50 g, and 22-month-old rats (the aged group, *n* = 40) weighing 550 ± 100 g (Tri-City Central Animal Laboratory, Research and Service Centre of the Medical University of Gdansk, Poland). For the duration of the experiment, the rats were maintained under standard conditions in the Certified Conventional Vivarium of the Department of Animal and Human Physiology at the University of Gdansk, and they were housed separately in polycarbonate cages (20 cm width, 37 cm length, 18 cm height). They were able to make eye contact and communicate via olfaction with each other, and their cages were on a 12 h light/dark cycle (lights on at 06:00) with the temperature maintained at 22 °C and the humidity at 50 ± 60%. Water and food were available ad libitum.

**Figure 8:**
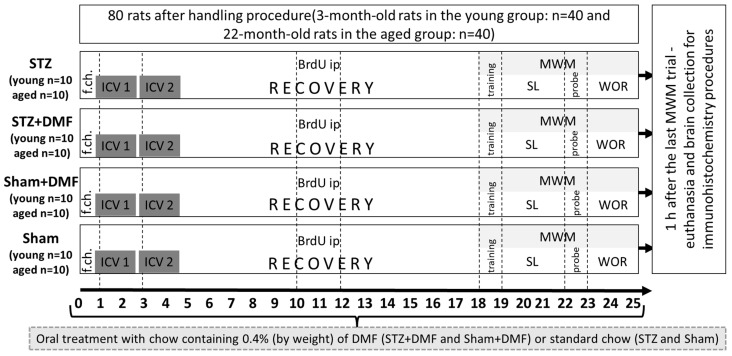
Diagram of experimental procedures and group assignments. Explanations: f.ch.—change in feed; ICV1/ICV2—intracerebroventricular injection (1—the first injection, 2—the second injection) of STZ (a total dose of 3 mg/kg divided into 2 injections to both lateral ventricles: 0.75 mg/kg STZ in 2 µL of citrate buffer per ventricle) or vehicle (citrate buffer 2 µL per ventricle); BrdU ip—intraperitoneal injection of 5-bromo-2’deoxyuridine (BrDU) at a dose of 50 mg/kg for three consecutive days; MWM—Morris water maze test; SL—spatial learning, the phase of acquisition of reference memory; probe—single trial without the platform (reference memory evaluation); WOR—working memory performance.

After a two-week habituation period, the animals in both age groups (young: *n* = 40; aged: *n* = 40) were randomly assigned to the following experimental groups: (1) STZ—rats with STZ-ICV, fed with standard chow (young: *n* = 10, aged: *n* = 10); (2) STZ+DMF—rats injected with STZ-ICV, fed with chow containing DMF (young: *n* = 10, aged: *n* = 10); (3) Sham+DMF—rats with an ICV of a vehicle (citrate buffer), fed with chow containing DMF (young: *n* = 10, aged: *n* = 10); Sham—rats with an ICV of a vehicle, fed with standard chow (young: *n* = 10, aged: *n* = 10). After being assigned to experimental groups, the animals were subjected to further procedures according to the diagram presented in Figure 8.

Changing the feed to DMF-containing (0.4% by weight) chow or standard chow was established one day before surgery (day 0 in Figure 8). The next day, the surgical ICV injection of STZ or the vehicle was carried out (day 1 in Figure 8). ICV injections were repeated after 2 days (day 3 in Figure 8) in order to divide the total dose of the STZ (3 mg/kg) into two injections, thereby minimizing mortality [17,18,77].

### 4.2. Dimethyl Fumarate Treatment

For the STZ+DMF and Sham+DMF animals, the standard chow was replaced with pellets containing 0.4% DMF by weight (Ssniff, Soest, Germany). It was confirmed that such a DMF concentration in the feed does not cause side effects, especially significant weight loss [17,18,78]. The DMF therapy was initiated the day before the first surgery (day 0 in Figure 8) and continued until the end of the experiment (day 25 in Figure 8). Both the feed intake and rats’ weight were measured daily.

### 4.3. Intracerebroventricular Injection of STZ

ICV injections of streptozotocin (STZ) or a vehicle (0.02 M citrate buffer pH 4.5) were performed according to our previous studies [17,18]. Briefly, rats under isoflurane anaesthesia were injected with STZ in a cumulative dose of 3 mg/kg over two injections on days 1 and 3 (1.5 mg/kg, dissolved in a 0.02 M citrate buffer, pH 4.5 each injection) with separate injections into each lateral ventricle (0.75 mg/kg in 2 µL of vehicle). The total dose of STZ was divided in order to reduce procedure-associated mortality according to our previous studies [17,18].

### 4.4. 5-bromo-2′-Deoxyuridine (BrdU) Administration

5-bromo-2′deoxyuridine (BrdU) is a synthetic analogue of thymidine, which integrates into DNA in the S phase of mitotic division, and it is commonly used as a marker of proliferating cells [13,27,33,79]. Seven days after the second ICV injection of the STZ/vehicle, all rats received three intraperitoneal (ip) injections of BrdU (Sigma Aldrich, St. Louis, MO, USA, #B5002) in a dose of 50 mg/kg each, with an interval of 24 h between them to maximise the labelling of dividing cells in the DG and OB (days 10–12 on Figure 8). For further procedures, the rats’ brains were collected 15 days after the first BrdU ip injection, which according to the literature (e.g., [13,80,81]) is enough time to develop a phenotype (neuronal or glial) of the newly formed cells, which allowed us to determine the level of adult neurogenesis in the above-mentioned regions of the rat brain in immunohistochemical procedures.

### 4.5. Test of Spatial Memory in the Morris Water Maze (MWM)

MWM testing was performed as described in detail in our previous study [17] with a few modifications. Briefly, the testing began with a training session 18 days after the initiation of DMF or control treatment (14 days after the second STZ or vehicle ICV injection). The spatial learning was tested on days 19, 20 and 21 and ended with a probe test (reference memory evaluation task with the platform removed) on day 22 of the procedure (Figure 8). Working memory was tested for the next three days (days 23–25). A training session (two trials with the visible platform) was conducted to exclude animals with motivational or sensory–motor deficits. In the first trial, a rat needed to find the platform within 60 s, otherwise it was gently guided towards the platform by the experimenter’s hand. After 5 min, a screening trial was performed. Spatial learning was tested in four 120 s trials per day with a 10 min intertrial interval for the next three days. During testing, the platform was hidden 1 cm below the surface of the water, and its position remained unchanged (centre of the NE quadrant—northeast quadrant of the pool). Each rat was placed in the pool on the opposite side of the platform: in the southwest (SW) quadrant facing the pool wall. Each trial ended when: (1) the animal found the platform within 120 s and remained on it for 5 s, in which case the animal was then removed from the pool to the home cage; or (2) the rat had not found the platform after 120 s, in which case the rat was navigated to the platform, left on it for 5 s and then removed from the pool and taken to the home cage. On the 22nd day of the experimental procedure, 24 h after the third session of spatial learning, the probe test (session 4) with the removed platform was performed. During this session, one 120 s trial was conducted for each rat (starting position in the SW quadrant). The working memory examination lasted from the fifth to the seventh day of the MWM (23rd to 25th day of the experimental procedure, Figure 8) and consisted of three sessions (one session daily) with four trials per session and a 10 min intertrial interval. The course of the testing procedure was the same as for sessions 1–3, except that the platform position was changed clockwise each day of sessions 5–7. Due to the change in the platform position from session to session, on the first trial of each day, the rat needed to scan the pool area to find the platform, and in the subsequent trials (2–4), the animal could use information about the platform location that was stored in their short-term memory, which was acquired in the previous trial.

The scores used to analyse the animal’s performance in spatial learning and working memory tasks included latency to reach the platform after releasing the animal into the pool and the percentage of the total distance travelled in the critical quadrant (CQ) of the pool (where the platform was located). The scores used to analyse the animal’s performance in the probe test (reference memory evaluation) included latency to reach the CQ after releasing the animal into the pool, the percentage of the total distance travelled in the CQ and the number of goal (the virtual contour of the removed platform) crossings. All the MWM parameters were measured with the use of a video-tracking digitising device (EthoVision XT, Noldus, Wageningen, The Netherlands).

### 4.6. Tissue Preparation

One hour after the last MWM trial of the seventh session (25th day of the procedure, Figure 8), the animals were euthanised with Morbital (2 mL/kg) and were perfused transcardially (via the left ventricle) with 200 mL of 0.9% saline followed by 200 mL of 4% paraformaldehyde in 0.1 M phosphate-buffered saline (PBS). The brains were postfixed and cryoprotected in a 30 % sucrose solution in PBS. Coronal brain sections (20 μm) were cut using a cryostat (CM 1850, Leica Biosystems, Nußloch, Germany). Three coronal brain sections per rat (*n* = 10 for each experimental group in both age groups) were collected for an immunofluorescent assessment of the adult neurogenesis parameters, and three coronal brain sections per rat (*n* = 10 for each experimental group in the young animals, *n* = 8 for aged STZ+DMF, *n* = 9–10 for aged STZ, *n* = 10 for both aged Sham groups) for the immunofluorescent assessment of the BDNF-containing cells were collected. Brain sections were obtained from two locations: 2.88 to 3.14 mm posterior from bregma for sections containing cells of the DG and CA1–CA3 areas of the hippocampus, and 5.64 mm anterior from bregma for sections containing cells of the OB according to the stereotaxic coordinates from the rat brain atlas [82].

### 4.7. Immunohistochemistry

In order to determine the proliferation (labelling with BrdU) and neuronal differentiation (labelling with DCX—doublecortin marker, which identifies immature neurons under development) and the level of newly formed immature neurons in the DG and OB, we used double immunofluorescence labelling on brain sections according to the method described in our previous study [17] with a few modifications. Briefly, free floating brain sections were immersed in a 2 M hydrochloric acid solution for 30 min at 37 °C (DNA denaturation), and then they were washed twice with phosphate-buffered saline (PBS). Next, the sections were blocked with 5% bovine serum albumin (BSA) in PBS with pH 7.4 containing 0.3% Triton X-100 for 3 h. After triple washing with PBS for 5 min, the sections were incubated with a mouse monoclonal primary anti-BrdU antibody (Pierce Antibodies, # OMA1-06200, 1:200) and rabbit polyclonal primary antidoublecortin antibody (Santa Cruz, Dallas, TX, USA, #sc-28939, 1:250) in PBS containing 0.3% Triton X-100 and 1% BSA at 4 °C for 48 h. After the incubation period and rinsing three times for 5 min in a 0.5M Tris buffer (pH = 7.4) to remove the excess unbound primary antibodies, the sections were incubated in the dark at 4 °C for 2 h in PBS containing an Alexa Fluor 546 donkey polyclonal secondary antimouse antibody (Thermo Fisher, Waltham, MA, USA, #A10036, 1:500) emitting orange light and an Alexa Fluor 488 donkey polyclonal secondary antirabbit antibody (Thermo Fisher, #A-21206, 1:500) emitting green light. The last step was washing the sections three times with PBS with a 0.5M Tris buffer and placing them on gelatine-coated slides using a sealing medium (Vectashield Mounting Medium, Vector, Burlingame, CA, USA, # H-1400) for fluorescence microscopy.

Neuroprotection was assessed by the BDNF-containing cells detection in both analysed neurogenic regions (the DG and OB) and in CA1–CA3 areas of the hippocampus. For BDNF immunofluorescence labelling, we performed an identical procedure as described above, except we used a rabbit polyclonal anti-BDNF antibody (Thermo Fisher, # PA1-18357, 1:1000) as the primary antibody and an Alexa Fluor 488 donkey polyclonal antirabbit antibody (Thermo Fisher, #A-21206, 1:500) as the secondary antibody.

### 4.8. Quantification of Labelled Cells

Prepared slides of the BrdU+DCX- and BDNF-labelled brain sections were analysed with a fluorescence microscope (Zeiss Axio Scope A1) equipped with a standard Cy-3 filter with the use of Axio Vision software. Digital images of the sections containing cells of the DG and CA1–CA3 areas of the hippocampus and OB were obtained. For the BrdU+DCX-labelled brain sections, two images were taken—the first with an orange (BrdU detection) and the second with a green (DCX detection) fluorescence filter. The third image was obtained by superimposing two previously taken images, which enabled the detection of cells containing both fluorochromes. For the BDNF-labelled brain sections, one image was taken with a green fluorescence filter. The borders of the brain structures were determined according to the Paxinos and Watson atlas [82]. A calibrated frame for cell counting (0.01 mm^2^) was imposed on the photographs in the software, and labelled cells were identified and counted by an observer blinded to the experimental group allocation. For the level of newly formed immature neurons, the percentage of double-labelled cells (containing BrdU and DCX) were determined from the sum of all fluorescent cells.

### 4.9. Data Analysis

The data are expressed as the mean ± SEM. Statistical analyses of spatial learning and working memory performance in the MWM test involved a repeated measures four-way ANOVA with the sAD-like model (STZ_Sham), DMF therapy (DMF_Standard chow) and age (Young_Aged) as the between-subjects factors, and the day or trial of the test as the within-subject factor, followed by Tukey’s post-hoc test. Statistical analyses of the reference memory performance in the MWM test and immunohistochemistry data involved a three-way ANOVA with the sAD-like model (STZ_Sham), DMF therapy (DMF_Standard chow) and age (Young_Aged) factors, followed by Tukey’s post-hoc test. The level of significance was set at *p* < 0.05. The statistical analyses were performed with the Statistica 12 (Statsoft) software.

## 5. Conclusions

Our results indicated that dimethyl fumarate alleviates the impairment of spatial cognition and adult neurogenesis in the dentate gyrus and olfactory bulb, as well as a decline in brain-derived neurotrophic factor expression in the hippocampus of young and aged rats subjected to an intracerebroventricular injection of streptozotocin. Advanced age adversely affected all these related parameters both in the control rats and in the streptozotocin-induced model of Alzheimer’s disease. Our findings suggest that DMF, due to its beneficial effect on the formation of new neurons and BDNF-related neuroprotection, may be considered a promising new therapeutic agent in reducing and/or delaying the onset of symptoms in human sAD.

## Figures and Tables

**Table 1:** Analysis of DMF-containing chow or standard chow intake, daily DMF dose and body weight changes during the experiment (data are expressed as mean ± SEM).

Experimental and Age Group	STZ+DMF Young	Sham+DMF Young	STZ+DMF Aged	Sham+DMF Aged
**Parameter**				
Initial body weight (g) ^1^	337.30 ± 9.91	346.70 ± 8.93	505.80 ± 8.33	548.10 ± 15.83
Final body weight (g) ^2^	319.90 ± 10.86	344.50 ± 10.15	465.00 ± 8.16	503.00 ± 15.68
Daily chow intake (g) ^3^	3.21 ± 0.29	3.52 ± 0.25	5.74 ± 0.60	5.80 ± 0.40
Daily DMF intake (mg) ^4^	12.83 ± 1.14	14.10 ± 1.00	22.98 ± 2.38	23.18 ± 1.59
Daily DMF dose (mg/kg) ^5^	40.14 ± 3.51	41.02 ± 2.80	49.36 ± 5.06	46.63 ± 3.68
Changes in body weight (%) ^6^	5.23 ± 0.88	0.65 ± 1.26	8.06 ± 0.73	8.28 ± 0.51
**Experimental and Age Group**	**STZ Young**	**Sham Young**	**STZ Aged**	**Sham Aged**
**Parameter**				
Initial body weight (g) ^1^	356.20 ± 7.80	341.00 ± 6.93	521.60 ± 8.05	539.40 ± 13.15
Final body weight (g) ^2^	329.60 ± 11.08	332.30 ± 11.26	509.10 ± 8.12	515.70 ± 7.50
Daily chow intake (g) ^3^	5.50 ± 0.68	6.53 ± 1.00	5.82 ± 0.57	6.15 ± 0.54
Changes in body weight (%) ^6^	5.28 ± 0.82	4.77 ± 0.61	4.53 ± 0.89	3.27 ± 0.83

Explanations: 1—average body weight of rats at the beginning of the experiment; 2—average body weight of rats on 25th day of the experiments; 3—calculated by total chow intake over 25 days; 4—calculated by daily chow intake and DMF concentration in chow (0.4% by weight); 5—calculated by daily DMF intake and final body weight of rats; 6—calculated by the difference between final and initial body weight of the rats in relation to the initial body weight.

**Table 2:** Spatial learning (acquisition of long-term memory) evaluated by Morris water maze (MWM) test (F values of ANOVA analysis).

Factors	df	Latency to Reach the Platform (s)	% of Distance in CQ
**Spatial Learning (sessions 1–3)**			
(1) sAD-like model (STZ_Sham)	1, 216	194.83 #	123.83 #
(2) Therapy (DMF_Stand)	1, 216	64.44 &	78.42 &
(3) Age (Young_Aged)	1, 216	88.82 &	54.59 &
(4) Day (1_2_3)	2, 216	49.31 &	15.34 &
**Interactions:**			
sAD-like model × Therapy		45.68 &	82.40 &
sAD-like model × Age		8.96 *	4.61 *
Therapy × Age		7.61 *	0.06
sAD-like model × Day		3.39 *	1.22
Therapy × Day		4.58 *	0.33
Age × Day		4.02 *	5.90 *
1 × 2 × 3		6.18 *	0.80
1 × 2 × 4		3.98 *	3.63 *
1 × 3 × 4		2.16	3.81 *
2 × 3 × 4		3.42 *	0.09
1 × 2 × 3 × 4		1.34	0.72

Explanations: #—*p* < 0.001; &—*p* < 0.01; *—*p* < 0.05.

**Table 3:** Reference memory performance evaluated by Morris water maze (MWM) test (F values of ANOVA analysis).

Factors	df	Latency to Reach the CQ (s)	% of Distance in CQ	Number of Goal Crossings
**Reference Memory (session 4—probe test)**				
(1) sAD-like model (STZ_Sham)	1, 72	86.84 #	65.41 #	18.42 &
(2) Therapy (DMF_Stand)	1, 72	16.68 &	34.23 &	5.52 *
(3) Age (Young_Aged)	1, 72	28.49 &	51.99 #	4.42 *
**Interactions:**				
sAD-like model × Therapy		11.26 *	18.86 &	6.13 *
sAD-like model × Age		4.23 *	9.04 *	1.19
Therapy × Age		4.89 *	8.38 *	0.48
1 × 2 × 3		2.12	4.68 *	0.41

Explanations: #—*p* < 0.001; &—*p* < 0.01; *—*p* < 0.05.

**Table 4:** Working memory performance evaluated by Morris water maze (MWM) test (F values of ANOVA analysis).

Factors	df	Latency to Reach the Platform (s)	% of Distance in CQ
**Working Memory (sessions 5–7)**			
(1) sAD-like model (STZ_Sham)	1, 288	154.56 #	198.98 #
(2) Therapy (DMF_Stand)	1, 288	159.27 #	142.75 #
(3) Age (Young_Aged)	1, 288	182.03 #	162.89 #
(4) Trial (1_2_3_4)	3, 288	81.46 &	136.11 #
**Interactions:**			
sAD-like model × Therapy		48.39 &	27.24 &
sAD-like model × Age		16.16 *	0.74
Therapy × Age		8.41 *	9.10 *
sAD-like model × Trial		17.84 *	14.71 *
Therapy × Trial		8.15 *	7.39 *
Age × Trial		7.45 *	8.93 *
1 × 2 × 3		0.01	2.68
1 × 2 × 4		8.80 *	4.16 *
1 × 3 × 4		0.59	4.58 *
2 × 3 × 4		1.32	2.06
1 × 2 × 3 × 4		2.36	1.84

Explanations: #—*p* < 0.001; &—*p* < 0.01; *—*p* < 0.05.

**Table 5:** Adult neurogenesis in the dentate gyrus of the hippocampus (DG) and in the olfactory bulb (OB) (F values of ANOVA analysis).

Factors	df	Number of BrdU^+^ Cells	Number of DCX^+^ Cells	% of Newly Formed Immature Neurons (BrdU+DCX)
**Neurogenesis in the DG**				
(1) sAD-like model (STZ_Sham)	1, 72	29.41 #	33.11 #	45.04 #
(2) Therapy (DMF_Stand)	1, 72	13.15 &	12.16 &	15.30 &
(3) Age (Young_Aged)	1, 72	29.58 #	20.68 #	24.49 #
**Interactions:**				
sAD-like model × Therapy		9.09 &	16.00 &	14.29 &
sAD-like model × Age		0.05	0.79	5.23 *
Therapy × Age		5.85 *	7.13 *	1.41
1 × 2 × 3		2.84	1.52	2.11
Neurogenesis in the OB				
(1) sAD-like model (STZ_Sham)	1, 72	37.01 #	79.55 #	49.24 #
(2) Therapy (DMF_Stand)	1, 72	4.96 *	58.78 #	21.16 #
(3) Age (Young_Aged)	1, 72	22.16 #	30.90 #	11.52 &
Interactions:				
sAD-like model × Therapy		9.35 *	17.28 &	21.79 &
sAD-like model × Age		11.95 &	10.07 &	3.93
Therapy × Age		7.52 *	7.37 *	0.23
1 × 2 × 3		2.53	3.36	3.79

Explanations: #—*p* < 0.001; &—*p* < 0.01; *—*p* < 0.05.

**Table 6:** Neuroprotection (BDNF-containing cells) in both neurogenic regions (DG and OB) and in CA1-CA3 areas of the hippocampus (F values of ANOVA analysis).

FACTORS	df	CA1	CA2	CA3	DG	OB
**Neuroprotection** **(BDNF-containing cells)**						
(1) sAD-like model (STZ_Sham)	1, 69	45.47 #	43.36 #	57.60 #	36.14 #	34.41 #
(2) Therapy (DMF_Stand)	1, 69	19.01 &	11.07 *	5.87 *	11.77 *	4.16 *
(3) Age (Young_Aged)	1, 69	22.56 #	35.44 #	23.54 &	26.48 &	27.70 &
**Interactions:**						
sAD-like model × Therapy		4.55 *	8.76 *	10.92 *	10.07 *	14.36 &
sAD-like model × Age		1.31	0.11	0.01	0.02	0.09
Therapy × Age		0.12	0.99	0.01	0.01	0.77
1 × 2 × 3		0.42	0.13	1.20	0.05	0.63

Explanations: #—*p* < 0.001; &—*p* < 0.01; *—*p* < 0.05.

## Abbreviations

Aβ	amyloid beta protein
AD	Alzheimer’s disease
ANOVA	analysis of variance
BDNF	brain-derived neurotrophic factor
BrdU	5-bromo-2′-deoxyuridine
CMI	mild cognitive impairment
CQ	critical quadrant (quadrant of the maze with the platform) in the Morris water maze
DCX	doublecortin
DG	dentate gyrus of the hippocampus
DMF	dimethyl fumarate
fAD	familial Alzheimer’s disease
GSK3β	Glycogen synthase kinase β
ICV	intracerebroventricular injection
ip	intraperitoneal injection
MWM	Morris water maze test
NF-κB	nuclear factor kappa-light-chain-enhancer of activated B cells
Nrf2	nuclear factor erythroid 2-related factor 2
OB	olfactory bulb
PBS	phosphate-buffered saline
RRMS	relapsing–remitting multiple sclerosis
sAD	sporadic Alzheimer’s disease
SEM	standard error of the mean
SGZ	subgranular zone of the dentate gyrus
STZ	streptozotocin
SVZ	subventricular zone
